# Genome sequence of the chemoheterotrophic soil bacterium *Saccharomonospora cyanea* type strain (NA-134^T^)

**DOI:** 10.4056/sigs.4207886

**Published:** 2013-09-30

**Authors:** Jan P. Meier-Kolthoff, Megan Lu, Marcel Huntemann, Susan Lucas, Alla Lapidus, Alex Copeland, Sam Pitluck, Lynne A. Goodwin, Cliff Han, Roxanne Tapia, Gabriele Pötter, Miriam Land, Natalia Ivanova, Manfred Rohde, Markus Göker, John C. Detter, Tanja Woyke, Nikos C. Kyrpides, Hans-Peter Klenk

**Affiliations:** 1 Leibniz Institute DSMZ – German Collection of Microorganisms and Cell Cultures, Braunschweig, Germany; 2 Los Alamos National Laboratory, Bioscience Division, Los Alamos, New Mexico, USA; 3 DOE Joint Genome Institute, Walnut Creek, California, USA; 4 Theodosius Dobzhansky Center for Genome Bionformatics, St. Petersburg State University, St. Petersburg, Russia; 5 Algorithmic Biology Lab, St. Petersburg Academic University, St.Petersburg, Russia; 6 Oak Ridge National Laboratory, Oak Ridge, Tennessee, USA; 7 HZI – Helmholtz Centre for Infection Research, Braunschweig, Germany

**Keywords:** draft genome, aerobic, chemoheterotrophic, Gram-positive, vegetative and aerial mycelia, spore-forming, non-motile, soil bacterium, *Pseudonocardiaceae*, CSP 2010

## Abstract

*Saccharomonospora cyanea* Runmao *et al*. 1988 is a member of the genus *Saccharomonospora* in the family *Pseudonocardiaceae* that is moderately well characterized at the genome level thus far. Members of the genus *Saccharomonospora* are of interest because they originate from diverse habitats, such as soil, leaf litter, manure, compost, surface of peat, moist, over-heated grain, and ocean sediment, where they probably play a role in the primary degradation of plant material by attacking hemicellulose. Species of the genus *Saccharomonospora* are usually Gram-positive, non-acid fast, and are classified among the actinomycetes. *S. cyanea* is characterized by a dark blue (= cyan blue) aerial mycelium. After *S. viridis*, *S. azurea*, and *S. marina*, *S. cyanea* is only the fourth member in the genus for which a completely sequenced (non-contiguous finished draft status) type strain genome will be published. Here we describe the features of this organism, together with the draft genome sequence, and annotation. The 5,408,301 bp long chromosome with its 5,139 protein-coding and 57 RNA genes was sequenced as part of the DOE funded Community Sequencing Program (CSP) 2010 at the Joint Genome Institute (JGI).

## Introduction

Strain NA-134^T^ (= DSM 44106 = ATCC 43724 = NBRC 14841) is the type strain of the species *Saccharomonospora cyanea* [1], one out of currently nine members in the genus *Saccharomonospora* [2]. The strain was originally isolated from a soil sample collected from Guangyun, Sichuan, China [1]. The genus name *Saccharomonospora* was derived from the Greek words for *sakchâr*, sugar, *monos*, single or solitary, and *spora*, a seed or spore, meaning the sugar(-containing) single-spored (organism) [3]; the species epithet was derived from the Latin adjective *cyanea*, dark blue, referring to the color of the aerial mycelium [1]. *S. cyanea* and the other type strains of the genus *Saccharomonospora* were selected for genome sequencing in a DOE Community Sequencing Project (CSP 312) at Joint Genome Institute (JGI), because members of the genus (which originate from diverse habitats such as soil, leaf litter, manure, compost, surface of peat, moist, over-heated grain and ocean sediment) are supposed to play a role in the primary degradation of plant material by attacking hemicellulose. This expectation was underpinned by the results of the analysis of the genome of *S. viridis* [4], one of the recently sequenced GEBA genomes [5]. The *S. viridis* genome, which was the first sequenced genome from a member of the genus *Saccharomonospora*, contained an unusually large number of genes, 24, for glycosyl hydrolases (GH) belonging to 14 GH families, which were identified in the Carbon Active Enzyme Database [6]. Hydrolysis of cellulose and starch were also reported for other members of the genus (that are included in CSP 312), such as *S. marina* [7], *S. halophila* [8], *S. saliphila* [9], *S. paurometabolica* [10], and *S. xinjiangensis* [11]. Here we present a summary classification and a set of features for *S. marina* NA-134^T^, together with the description of the genomic sequencing and annotation.

## Classification and features

A representative genomic 16S rRNA sequence of strain NA-134^T^ was compared using NCBI BLAST [12,13] under default settings (e.g., considering only the high-scoring segment pairs (HSPs) from the best 250 hits) with the most recent release of the Greengenes database [14] and the relative frequencies of taxa and keywords (reduced to their stem [15]) were determined, weighted by BLAST scores. The most frequently occurring genera were *Saccharomonospora* (72.4%), *Prauserella* (11.3%), *Kibdelosporangium* (6.7%), *Amycolatopsis* (4.3%) and *Actinopolyspora* (3.0%) (101 hits in total). Regarding the two hits to sequences from members of the species, the average identity within HSPs was 99.9%, whereas the average coverage by HSPs was 99.8%. Regarding the 47 hits to sequences from other members of the genus, the average identity within HSPs was 97.1%, whereas the average coverage by HSPs was 98.5%. Among all other species, the one yielding the highest score was *S. xinjiangensis* (AJ306300), which corresponded to an identity of 98.7% and an HSP coverage of 100%. (Note that the Greengenes database uses the INSDC (= EMBL/NCBI/DDBJ) annotation, which is not an authoritative source for nomenclature or classification.) The highest-scoring environmental sequence was FN667150 ('stages composting process full scale municipal waste compost clone FS1575'), which showed an identity of 99.6% and an HSP coverage of 97.9%. The most frequently occurring keywords within the labels of all environmental samples which yielded hits were 'skin' (26.6%), 'nare' (10.5%), 'fossa' (5.3%), 'forearm, volar' (4.5%) and 'human' (4.4%) (149 hits in total), and show no fit to the habitats from which the validly named members of the genus were isolated. The most frequently occurring keywords within the labels of those environmental samples which yielded hits of a higher score than the highest scoring species were 'compost' (25.0%) and 'full, municip, process, scale, stage, wast' (12.5%) (1 hit in total).

**Figure 1:**
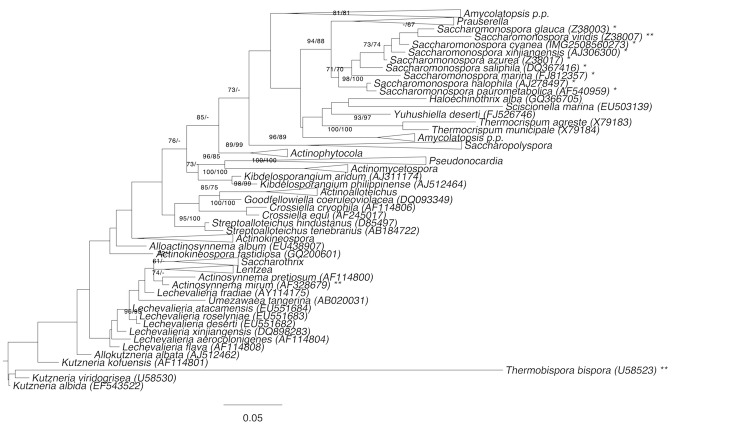
Phylogenetic tree highlighting the position of *S. cyanea* relative to the type strains of the other species within the family *Pseudonocardiaceae*. The tree was inferred from 1,371 aligned characters [16,17] of the 16S rRNA gene sequence under the maximum likelihood (ML) criterion [18]. Rooting was done initially using the midpoint method [19] and then checked for its agreement with the current classification (Table 1). The branches are scaled in terms of the expected number of substitutions per site. Numbers adjacent to the branches are support values from 600 ML bootstrap replicates [20] (left) and from 1,000 maximum-parsimony bootstrap replicates [21] (right) if larger than 60%. Lineages with type strain genome sequencing projects registered in GOLD [22] are labeled with one asterisk, those also listed as 'Complete and Published' with two asterisks [4,23,24] (*S. azurea* [25] and *S. marina* [26] miss their second asterisk due to very recent publication). *Actinopolyspora iraqiensis* Ruan *et al*. 1994 was ignored in the tree, because a proposal for the transfer of this species to the genus *Saccharomonospora* [27] was recently rejected on formal criteria [3].

Figure 1 shows the phylogenetic neighborhood of *S. cyanea* in a 16S rRNA based tree. The sequences of the three identical 16S rRNA gene copies in the genome differ by one nucleotide from the previously published 16S rRNA sequence (Z38018).

**Table 1:** Classification and general features of *S. cyanea* NA-134^T^ according to the MIGS recommendations [28] published by the Genome Standards Consortium [29].

**MIGS ID**	**Property**	**Term**	**Evidence code**
	Current classification	Domain *Bacteria*	TAS [30]
		Phylum *Actinobacteria*	TAS [31]
		Class *Actinobacteria*	TAS [32]
		Subclass *Actinobacteridae*	TAS [32,33]
		Order *Actinomycetales*	TAS [32-35]
		Suborder *Pseudonocardineae*	TAS [32,33,36]
		Family *Pseudonocardiaceae*	TAS [36] [32,33,36] [32,33,36] [[36] [32,33,36-38]
		Genus *Saccharomonospora*	TAS [34,39]
		Species *Saccharomonospora cyanea*	TAS [1]
		Type-strain NA-134	TAS [1]
	Gram stain	positive	NAS
	Cell shape	variable, substrate and aerial mycelia	TAS [1]
	Motility	non-motile	TAS [1]
	Sporulation	small, non-motile spores with warty surface; single and mostly from aerial mycelium	TAS [1]
	Temperature range	mesophile, 24-40°C	TAS [1]
	Optimum temperature	28-37°C	TAS [1]
	Salinity	grows well in up to 10% (w/v) NaCl	TAS [1]
MIGS-22	Oxygen requirement	aerobic	TAS [1]
	Carbon source	pentoses, hexoses, but not D-glucose	TAS [1]
	Energy metabolism	chemoheterotrophic	NAS
MIGS-6	Habitat	soil	TAS [1]
MIGS-15	Biotic relationship	free living	TAS [1]
MIGS-14	Pathogenicity	none	NAS
	Biosafety level	1	TAS [40]
MIGS-23.1	Isolation	soil	TAS [1]
MIGS-4	Geographic location	Guangyan City, Sichuan, China	TAS [1]
MIGS-5	Sample collection time	1988 or before	NAS
MIGS-4.1	Latitude	32.450	TAS [1]
MIGS-4.2	Longitude	105.843	TAS [1]
MIGS-4.3	Depth	not reported	
MIGS-4.4	Altitude	about 40 m	NAS

Evidence codes - TAS: Traceable Author Statement (i.e., a direct report exists in the literature); NAS: Non-traceable Author Statement (i.e., not directly observed for the living, isolated sample, but based on a generally accepted property for the species, or anecdotal evidence). These evidence codes are from of the Gene Ontology project [41].

**Figure 2:**
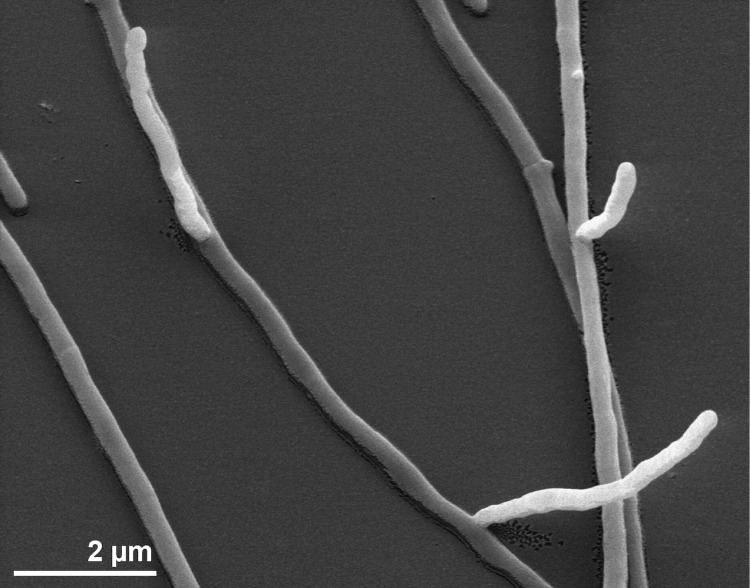
Scanning electron micrograph of *S. cyanea* NA-134^T^

Cells of strain NA-134^T^ form an irregularly branched, non-fragmenting, vegetative mycelium of 0.2 to 0.4 μm diameter (Figure 2) [1]. The aerial mycelium had a diameter of 0.3 to 0.6 μm and formed more sessile spores than the substrate mycelium [1]. Spores are non-motile, small, and oval to ellipsoid with warty surface [1]. The growth range of strain NA-134^T^ spans from 24°C to 40°C, with an optimum at 28°C to 37°C [1]. Strain NA-134^T^ grows well in up to 10% NaCl, but not in 15% NaCl [1]. Substrates used by the strain are summarized in detail in the strain description [1].

### Chemotaxonomy

The cell wall of strain NA-134^T^ are of type IV, containing *meso*-diaminopimelic acid, with type A whole-cell sugar pattern (galactose and arabinose present) [1]. The fatty acids spectrum is dominated by saturated penta- to heptadecanoic acids: C_17:1 cis-9_ (17.0%), *anteiso-*C_17:0_ (13.0%), *iso-*C_16:0_ (12.0%), C_17:0_ (11.5%), C_15:0_ (7.0%), *iso-*C_16:0 2OH_ (7.0%), C_16:1 cis-9_ (6.0%), C_16:0_ (5.0%), *iso-*C_15:0_ (3.0%), and *anteiso-*C_15:0_ (2.0%) [42].

## Genome sequencing and annotation

### Genome project history

This organism was selected for sequencing as part of the DOE Joint Genome Institute Community Sequencing Program (CSP) 2010, CSP 312, “Whole genome type strain sequences of the genus *Saccharomonospora* – a taxonomically troubled genus with bioenergetic potential”. The genome project is deposited in the Genomes On Line Database [22] and the complete genome sequence is deposited in GenBank. Sequencing, finishing and annotation were performed by the DOE Joint Genome Institute (JGI) using state of the art sequencing technology [43]. A summary of the project information is shown in Table 2.

**Table 2:** Genome sequencing project information

**MIGS ID**	**Property**	**Term**
MIGS-31	Finishing quality	Non-contiguous finished
MIGS-28	Libraries used	Three genomic libraries: one 454 pyrosequence standard library, one 454 PE library (12 kb insert size), one Illumina library
MIGS-29	Sequencing platforms	Illumina GAii, 454 GS FLX Titanium
MIGS-31.2	Sequencing coverage	1,005.1 × Illumina; 8.6 × pyrosequence
MIGS-30	Assemblers	Newbler version 2.3, Velvet version 1.0.13, phrap version SPS - 4.24
MIGS-32	Gene calling method	Prodigal, GenePRIMP
	INSDC ID	CM001440, AHLY00000000.1
	GenBank Date of Release	February 3, 2012
	GOLD ID	Gi07556
	NCBI project ID	61997
	Database: IMG	2508501013
MIGS-13	Source material identifier	DSM 44106
	Project relevance	Bioenergy and phylogenetic diversity

### Growth conditions and DNA isolation

Strain NA-134^T^, DSM 44106, was grown in DSMZ medium 3 (*Azotobacter* Medium) [44] at 28°C. DNA was isolated from 0.5-1 g of cell paste using Jetflex Genomic DNA Purification Kit (GENOMED 600100) following the standard protocol as recommended by the manufacturer with the following modifications: extended cell lysis time (60 min.) with additional 30µl achromopeptidase, lysostaphin, mutanolysin; proteinase K was applied in 6-fold the supplier recommended amount for 60 min. at 58°C. The purity, quality and size of the bulk gDNA preparation were according to DOE-JGI guidelines and routine protocols by the DNA Bank Network [45]. DNA is available through the DNA Bank Network [46].

### Genome sequencing and assembly

The genome was sequenced using a combination of Illumina and 454 sequencing platforms. All general aspects of library construction and sequencing can be found at the JGI website [47]. Pyrosequencing reads were assembled using the Newbler assembler (Roche). The initial Newbler assembly consisting of 148 contigs in one scaffold was converted into a phrap [48] assembly by making fake reads from the consensus, to collect the read pairs in the 454 paired end library. Illumina GAii sequencing data (5,624.1 Mb) were assembled with Velvet [49] and the consensus sequences were shredded into 1.5 kb overlapped fake reads and assembled together with the 454 data. The 454 draft assembly was based on 103.5 Mb 454 draft data and all of the 454 paired end data. Newbler parameters are -consed -a 50 -l 350 -g -m -ml 20. The Phred/Phrap/Consed software package [48] was used for sequence assembly and quality assessment in the subsequent finishing process. After the shotgun stage, reads were assembled with parallel phrap (High Performance Software, LLC). Possible mis-assemblies were corrected with gapResolution [47], Dupfinisher [50], or sequencing cloned bridging PCR fragments with subcloning. Gaps between contigs were closed by editing in Consed, by PCR and by Bubble PCR primer walks (J.-F. Chang, unpublished). A total of 157 additional reactions were necessary to close gaps and to raise the quality of the finished sequence. Illumina reads were also used to correct potential base errors and increase consensus quality using a software Polisher developed at JGI [51]. The error rate of the completed genome sequence is less than 1 in 100,000. Together, the combination of the Illumina and 454 sequencing platforms provided 1,013.7 × coverage of the genome. The final assembly contained 366,256 pyrosequence and 71,412,890 Illumina reads.

### Genome annotation

Genes were identified using Prodigal [52] as part of the DOE-JGI [53] genome annotation pipeline, followed by a round of manual curation using the JGI GenePRIMP pipeline [54]. The predicted CDSs were translated and used to search the National Center for Biotechnology Information (NCBI) non-redundant database, UniProt, TIGRFam, Pfam, PRIAM, KEGG, COG, and InterPro databases. Additional gene prediction analysis and functional annotation was performed within the Integrated Microbial Genomes - Expert Review (IMG-ER) platform [55].

## Genome properties

**Figure 3:**
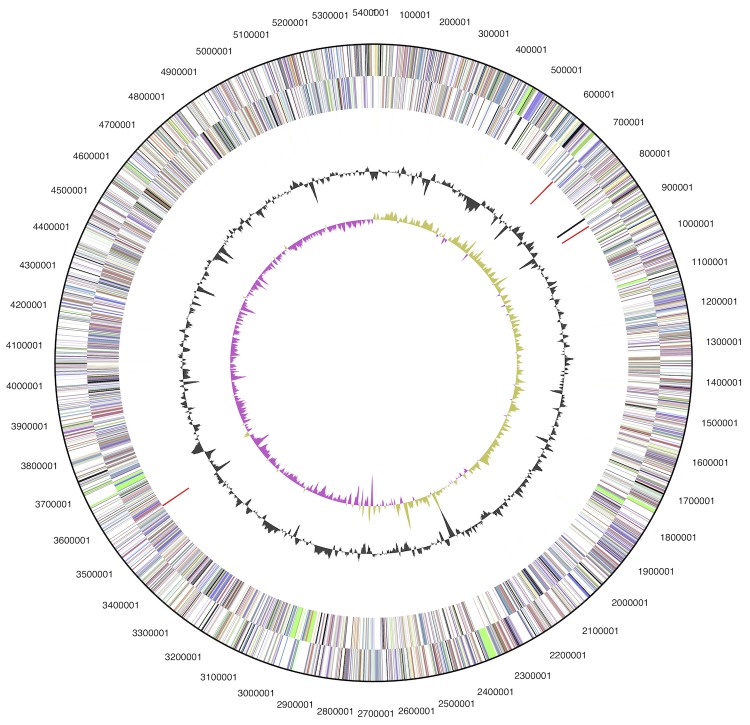
Graphical map of the chromosome. From outside to the center: Genes on forward strand (color by COG categories), Genes on reverse strand (color by COG categories), RNA genes (tRNAs green, rRNAs red, other RNAs black), GC content, GC skew (purple/olive).

The genome consists of a 5,408,301 bp long circular chromosome with a 69.7% G+C content (Table 3 and Figure 3). Of the 5,196 genes predicted, 5,139 were protein-coding genes, and 57 RNAs; 93 pseudogenes were also identified. The majority of the protein-coding genes (74.7%) were assigned a putative function while the remaining ones were annotated as hypothetical proteins. The distribution of genes into COGs functional categories is presented in Table 4.

**Table 3:** Genome Statistics

**Attribute**	Value	% of Total
Genome size (bp)	5,408,301	100.00%
DNA coding region (bp)	4,926,834	91.10%
DNA G+C content (bp)	3,771,475	69.74%
Number of replicons	1	
Extrachromosomal elements	0	
Total genes	5,196	100.00%
RNA genes	57	1.10%
rRNA operons	3	
tRNA genes	47	0.90%
Protein-coding genes	5,139	98.90%
Pseudo genes	93	1.79%
Genes with function prediction (proteins)	3,880	74.67%
Genes in paralog clusters	2,852	54.89%
Genes assigned to COGs	3,834	73.79%
Genes assigned Pfam domains	4,014	77.25%
Genes with signal peptides	1,512	29.10%
Genes with transmembrane helices	1,206	23.21%
CRISPR repeats	0	

**Table 4:** Number of genes associated with the general COG functional categories

**Code**	**value**	**%age**	**Description**
J	184	4.3	Translation, ribosomal structure and biogenesis
A	1	0.0	RNA processing and modification
K	512	11.8	Transcription
L	182	4.2	Replication, recombination and repair
B	2	0.1	Chromatin structure and dynamics
D	34	0.8	Cell cycle control, cell division, chromosome partitioning
Y	0	0.0	Nuclear structure
V	79	1.8	Defense mechanisms
T	209	4.8	Signal transduction mechanisms
M	174	4.0	Cell wall/membrane biogenesis
N	5	0.1	Cell motility
Z	0	0.0	Cytoskeleton
W	0	0.0	Extracellular structures
U	35	0.8	Intracellular trafficking and secretion, and vesicular transport
O	130	3.0	Post-translational modification, protein turnover, chaperones
C	274	6.3	Energy production and conversion
G	327	7.6	Carbohydrate transport and metabolism
E	341	7.9	Amino acid transport and metabolism
F	96	2.2	Nucleotide transport and metabolism
H	202	4.7	Coenzyme transport and metabolism
I	213	4.9	Lipid transport and metabolism
P	210	4.9	Inorganic ion transport and metabolism
Q	196	4.5	Secondary metabolites biosynthesis, transport and catabolism
R	588	13.6	General function prediction only
S	330	7.6	Function unknown
-	1,362	26.2	Not in COGs

## Insights into the genome sequence

### Comparative genomics

The phylum *Actinobacteria* is one of the most species-rich phyla in the domain *Bacteria* [31]. As of today the phylum contains the following ten orders, *Acidimicrobiales, Actinomycetales, Bifidobacteriales, Coriobacteriales, Euzebyales, Gaiellales, Nitriliruptorales, Rubrobacterales, Solirubrobacterales, Thermoleophilales*, with a total of 58 families [3]. Among these, the family *Pseudonocardiaceae* holds the genus *Saccharomonospora,* with 5 out of the 9 type strains for the member species having already completely sequenced genomes; the remaining 4 type strains have yet unpublished draft genome sequences according to the Genomes On Line Database (GOLD) [22].

Here we present a brief comparative genomics comparison of *S. cyanea* with a selection of its closest phylogenetic neighbors that have already published genome sequences (according to Figure 1): *S. viridis* [4], *S. azurea* [25] and *S. marina* [26].

The genomes of the four sequenced *Saccharomonospora* type strains differ significantly in their size, *S. cyanea* having 5.4 Mbp, *S. viridis* 4.3 Mbp, *S. azurea* 4.8 Mbp and *S. marina* 6.0 Mbp and their total number of genes, 5,196, 3,962, 4,530 and 5,784, respectively.

An estimate of the overall similarity between *S. cyanea*, on the one hand, and *S. viridis, S. azurea and S. marina*, on the other hand, was generated with the Genome-to-Genome Distance Calculator (GGDC) [56-58]. This system calculates the distances by comparing the genomes to obtain HSPs (high-scoring segment pairs) and interfering distances *via* a set of formulas (1, HSP length / total length; 2, identities / HSP length; 3, identities / total length). For convenience the GGDC also reports model-based DDH estimates along with their confidence intervals [58]. Table 5 shows the results of the pairwise comparison.

**Table 5:** Pairwise comparison of *S. cyanea* with *S. viridis, S. azurea* and *S. marina* using the GGDC (Genome-to-Genome Distance Calculator).

		**HSP length /** **total length [%]**	**Identities /** **HSP length [%]**	**Identities /** **total length [%]**
*S. cyanea*	*S. azurea*	71	85	61
*S. cyanea*	*S. marina*	28	79	22
*S. cyanea*	*S. viridis*	55	82	45

The comparison of *S. cyanea* with *S. azurea* reached the highest scores using the GGDC, 71% of the average of genome length are covered with HSPs. The identity within the HSPs was 85%, whereas the identity over the whole genome was 61%. The lowest similarity scores were observed in the comparison of *S. cyanea* with *S. marina with* only 28% of the average of both genome lengths covered with HSPs. The identity within these HSPs was 79%, whereas the identity over the whole genome was only 22%.

With regard to *S. cyanea* and *S. azurea* the corresponding DDH estimates were below the 70% threshold under formulas 1-3 throughout: 52.6% (±3), 28.6% (±3) and 45.4% (±3). The DDH estimates confidence intervals are given in parentheses as provided by [58]. The remaining pairings resulted in even smaller DDH estimates (data not shown).

As expected, those distances relating HSP coverage (formula 1) and number of identical base pairs within HSPs to total genome length (formula 3) are higher between *S. cyanea* and *S. azurea* than between *S. cyanea* and *S. viridis* or *S. marina*, respectively. That the distances relating the number of identical base pairs to total HSP length (formula 2) behave differently indicates that the genomic similarities between all four type strain genomes are strongly restricted to more conserved sequences, a kind of saturation phenomenon [56].

In order to further compare the genomes of *S. cyanea, S. viridis, S. azurea* and *S. marina*, correlation values (Pearson coefficient) according to the similarity on the level of COG category, pfam and TIGRfam were calculated (see Table 6). The highest correlation value (0.97) was reached for *S. cyanea* and *S. azurea* on the level of pfam data; the correlation values on the basis of COG and TIGRfam data were only slightly smaller with 0.96 and 0.93, respectively. As a correlation value of 1 indicates the highest correlation, we can find a very high correlation between the genomes of *S. cyanea* and *S. azurea* considering the above data [55].

**Table 6:** Pearson's correlation coefficients according to the similarity on the level of Pfam, COG category and TIGRfam (in this order and separated by slashes).

	*S. cyanea*	*S. azurea*	*S. viridis*	*S. marina*
*S. cyanea*	1.00 / 1.00 / 1.00	-	-	-
*S. azurea*	0.97 / 0.96 / 0.93	1.00 / 1.00 / 1.00	-	-
*S. viridis*	0.95 / 0.90 / 0.87	0.96 / 0.93 / 0.90	1.00 / 1.00 / 1.00	-
*S. marina*	0.93 / 0.90 / 0.86	0.93 / 0.90 / 0.87	0.94 / 0.90 / 0.83	1.00 / 1.00 / 1.00

The comparison of the number of genes belonging to the different COG categories revealed only small differences in the genomes of *S. cyanea* and *S. azurea* with 0.4% deviation between the same COG categories on average. A slightly higher fraction of genes belonging in the categories transcription (*S. cyanea* 11.8%, *S. azurea* 10.6%), carbohydrate metabolism (*S. cyanea* 7.6%, *S. azurea* 7.0%), secondary catabolism (*S. cyanea* 4.5%, *S. azurea* 4.1%), defense mechanisms (*S. cyanea* 1.8%, *S. azurea* 1.6%), inorganic ion transport and metabolism (*S. cyanea* 4.9%, *S. azurea* 4.7%) and lipid transport (*S. cyanea* 4.9%, *S. azurea* 4.8%) were identified in *S. cyanea*. The gene count in further COG categories such as cell cycle control, cell motility, cell biogenesis, lipid metabolism, secondary catabolism, post-translational modification and signal transduction was also slightly increased in *S. cyanea* but differed at most by 5 genes. In contrast, a slightly smaller fraction of genes belonging in the categories posttranslational modification (*S. cyanea* 3.0%, *S. azurea* 3.6%), coenzyme metabolism (*S. cyanea* 4.7%, *S. azurea* 5.2%), amino acid metabolism (*S. cyanea* 7.9%, *S. azurea* 8.4%), replication system (*S. cyanea* 4.2%, *S. azurea* 4.7%), translation (*S. cyanea* 4.3%, *S. azurea* 4.6%), signal transduction (*S. cyanea* 4.8%, *S. azurea* 5.1%), energy production/conversion (*S. cyanea* 6.3%, *S. azurea* 6.6%), nucleotide transport (*S. cyanea* 2.2%, *S. azurea* 2.4%) and cell wall biogenesis (*S. cyanea* 4.0%, *S. azurea* 4.2%) were identified in *S. cyanea*. The remaining COG categories intracellular transport, cell cycle control, cell motility and RNA modification differed by not more than a single gene.

**Figure 4:**
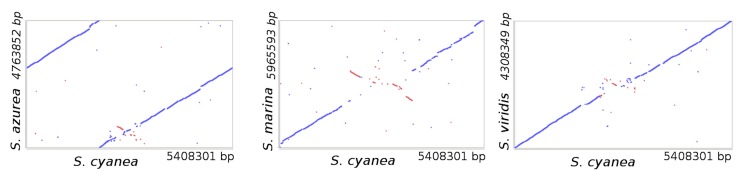
Synteny dot plot based on the genome sequences of *S. cyanea* vs. those of *S. viridis, S. azurea* and *S. marina*. Blue dots represent regions of similarity found on parallel strands and red dots show regions of similarity found on anti-parallel strands.

The synteny dot plots in Figure 4 shows nucleotide-based comparisons of the genomes of *S. cyanea* vs. *S. viridis, S. azurea* and *S. marina*. In most parts of the genomes a high degree of similarity becomes visible with only a little amount of indels. There exists a pronounced collinearity between the four genomes.

**Figure 5:**
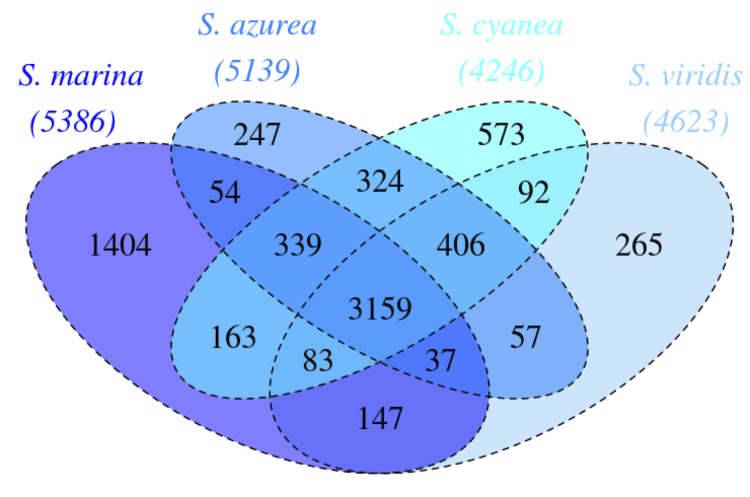
Venn-diagram depicting the intersections of protein sets (total numbers in parentheses) of *S. marina*, *S. azurea, S. cyanea* and *S. viridis*. The diagram was created with [59].

The Venn-diagram Figure 5 shows the number of shared genes between the completely sequenced and published genomes of *Saccharomonospora* type strains. All four genomes share a rather high fraction of 3,159 genes (59-74% of the genes, respectively) whereas only 247 (*S. azurea*, 5%) to 1,401 (*S. marina*, 26%) genes are unique for one genome in the genus. The genomes of *S. cyanea* and *S. azurea* contain the highest number (324) of pairwise shared genes, including many that encode hypothetical or unknown proteins (expectedly, due to the low level of functionally characterized genes in the genus), but also numerous transcriptional regulators (such as Sigma-70 and ATP-dependent transcriptional regulator) and transporters (such as TRAP transporters, arabinose efflux permeases, ABC-type sugar transport systems and Fe^3+^- transport systems, p-aminobenzoyl-glutamate transporter, 2-keto-3-deoxygluconate permease, Na^+^/H^+^ antiporter NhaD and related arsenite permeases, H^+^/gluconate symporter and related permeases). Surprisingly, these two genomes also share a suite of gas vesicle synthesis proteins.
